# Fractional Dynamics of Globally Slow Transcription and Its Impact on Deterministic Genetic Oscillation

**DOI:** 10.1371/journal.pone.0038383

**Published:** 2012-06-05

**Authors:** Kun Wei, Shilong Gao, Suchuan Zhong, Hong Ma

**Affiliations:** 1 College of Mathematics, Sichuan University, Chengdu, Sichuan Province, People’s Republic of China; 2 College of Life Sciences, Sichuan University, Chengdu, Sichuan Province, People’s Republic of China; National Research & Technology Council, Argentina

## Abstract

In dynamical systems theory, a system which can be described by differential equations is called a continuous dynamical system. In studies on genetic oscillation, most deterministic models at early stage are usually built on ordinary differential equations (ODE). Therefore, gene transcription which is a vital part in genetic oscillation is presupposed to be a continuous dynamical system by default. However, recent studies argued that discontinuous transcription might be more common than continuous transcription. In this paper, by appending the inserted silent interval lying between two neighboring transcriptional events to the end of the preceding event, we established that the running time for an intact transcriptional event increases and gene transcription thus shows slow dynamics. By globally replacing the original time increment for each state increment by a larger one, we introduced fractional differential equations (FDE) to describe such globally slow transcription. The impact of fractionization on genetic oscillation was then studied in two early stage models – the Goodwin oscillator and the Rössler oscillator. By constructing a “dual memory” oscillator – the fractional delay Goodwin oscillator, we suggested that four general requirements for generating genetic oscillation should be revised to be negative feedback, sufficient nonlinearity, sufficient memory and proper balancing of timescale. The numerical study of the fractional Rössler oscillator implied that the globally slow transcription tends to lower the chance of a coupled or more complex nonlinear genetic oscillatory system behaving chaotically.

## Introduction

The design and construction of genetic circuits are of great importance to the nascent field of synthetic biology [1]. A vital design principle for synthetic genetic circuits is to insure the capability of self-sustained oscillations for adapting the biological rhythms or environmental cycles. For example, the Goodwin oscillator which is considered to be the simplest genetic oscillator and the basis of the repressilators [1], [2] has been used as a minimal model to interpret the circadian rhythms occurring in gene’s negative autoregulation [3], [4]. Usually, one may expect to describe a basic genetic circuit by using a minimal dynamical model (with as few equations as possible), for the purpose of simplicity. The variables in such model represent the quantities of several key products in the circuit. However, as we have known, even the simplest genetic regulation includes complex intermediate processes like transcription, transportation of RNA, RNA splicing, RNA capping, translation, transportation of mature protein and other steps of post-translational modification. Therefore, a minimal model (sometimes with only a single equation) often lacks power to cover such complex intermediate processes in a regular timescale. This can be seen from the case that a Goodwin oscillator which requires an unrealistic high Hill coefficient (larger than 8) for the destabilization of a fixed point and generating limit cycle oscillations [5], [6].

However, by altering the timescale of gene transcription and introducing slow dynamics (e.g. considering the time lag in the protein transportation), one can readily obtain the desirable dynamical behaviors by using minimal models. A common method to achieve slow dynamics is introducing explicit time delay. In such way, sufficient time delay is considered to be one of the general requirements for sustained oscillations [7]. Another method is inserting an additional equation for lagging fast change in protein level, which plays a dynamical role similar to explicit time delays or to transport equations [8]. The two methods above display certain sorts of memory effects in gene transcription [7], [8]. These optimizations in timescale are designed merely for a specific intermediate product (e.g. protein) or for a specific intermediate biochemical step. We thus regard the above timescale changes as non-global.

In this paper, we would like to introduce the globally slow transcription which can be described by fractional differential equations (FDE). This idea is based on the recent evidence that discontinuous transcription may be more common than continuous transcription. In the discontinuous transcription case, by inserting silent intervals into neighboring transcriptional events which are presupposed to be continuous, and appending an inserted silent interval to the end of the preceding event, we aim to establish that the running time for an intact transcriptional event increases and the transcription system thus shows the property of globally slow transcription. Effects of such globally slow transcription are investigated in a minimal Goodwin oscillator and a Rössler oscillator, both of which are well-known in genetic regulation.

## Materials and Methods

### Definitions of Fractional Calculus and Memory Weighted via the Convolution Kernel

In spite of the existence of different definitions for the fractional derivatives, the fractional integral is the common foundation of fractional calculus. The notion of the left-side fractional integral operator with order 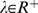 is in fact an extension of the Cauchy’s formula for repeated integrals which replaces the 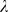-fold integrals of a function 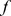 by a simple convolution:
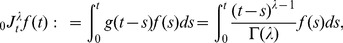
(1)where 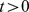, 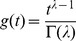, and 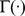 is the Gamma function. Moreover, under certain reasonable assumptions there exists 
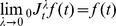
; we then obtain an identity operator denoted by [9]

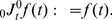
(2)Considering that in real world applications, the evolution of a general dynamical system governed by the principle of causality is apriori time-irreversible, we use the left-side integral/derivative operators and the initial time 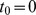 throughout this paper.

The derivative operator under the Caputo definition is expressed as follows [10]:

(a) If 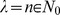, we have the integer order derivatives:
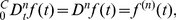
(3)where 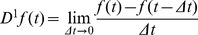, demonstrating the local property at a given time point 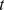. In particular, there exists 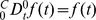.

(b) If 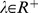 and 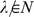, the left-side Caputo fractional derivative operator is represented by
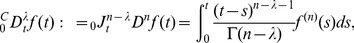
(4)where 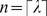 and 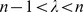. In particular, when 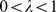, we have
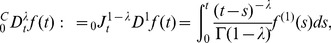
(5)(c) If 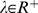, then
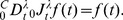
(6)From Eq. (3) and Eq. (4) we can see that the difference between the integer order derivative operator and the fractional (non-integer order) derivative operator is caused by the integral 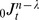 (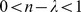) which endows the operator 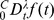 with the non-local property because the information of the entire integral interval 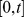 is involved. This is why we regard the fractional integral as the common foundation of fractional calculus.

**Figure 1:**
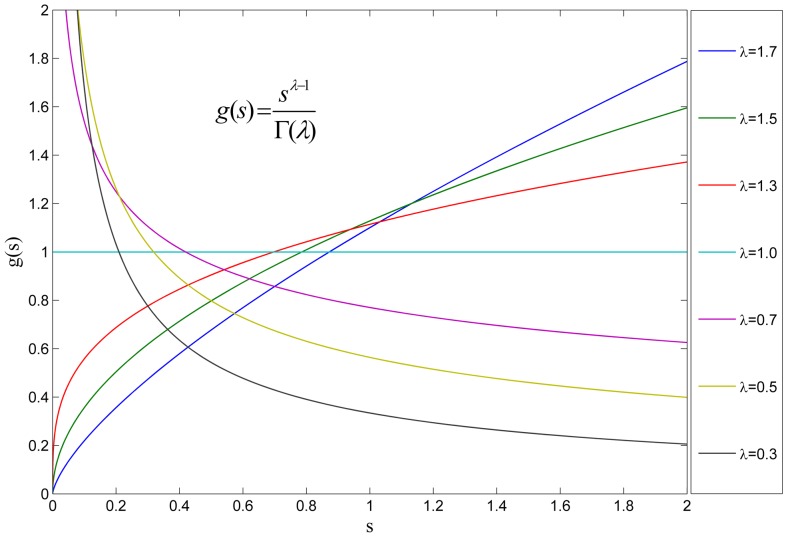
Graph of the weighting function 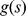 with intermediate values of 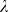. With 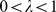, the curves demonstrate the property of fading memory, while in the case where 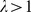, the memory of earlier time is enhanced while recent values of 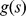 are attenuated.

In dynamical systems theory, memory effects are usually described by explicit non-local terms about the state variables. In Eq. (1), we know 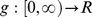. If 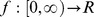, according to the commutative property of convolution, we have
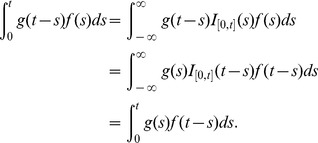
(7)Then, Eq. (1) can be written as



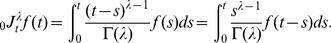
(8)It is obvious that the fractional integral Eq. (8) reflects a weighted average of delays 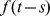 via a specific convolution kernel function 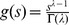. The values of the weighting function 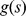 change with 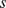 under different order 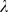 are illustrated in Fig. 1. In the circumstance where 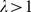, the memory is enhanced with 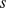 increasing, while states closed to present are given little weights. For 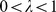, 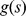 decreases with increasing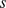, leading to the “fading memory” property with which the importance of the past state 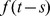 fades out. In this scenario, the larger values of 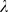 provide slower decay of 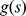. In the limit as 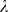 approaches zero, 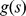 approaches the Dirac delta function, leading to the identity operator (zero order calculus). In contrast, in the limit as 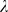 approaches 1, the weighting function 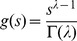 approaches the constant 1, providing same weights for all states.

### Transcriptional Discontinuity Builds the Link between Gene Transcription and Fractional Calculus

In early models of genetic regulation, the system of gene transcription is presupposed to be a continuous dynamical system and thus its long-time behavior can be described by using an ordinary differential equation (ODE) which contains a Michaelis-Menten (MM) mRNA synthesis term. For example, the one-variable Goodwin model is
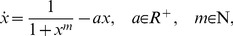
(9)where 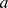 is the degradation coefficient and 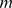 is Hill coefficient [6], [11]. By examining the two-variable extension of Eq. (9):



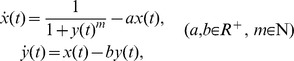
and the three-variable extension:



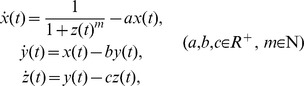
Griffith [6] found that the addition of intermediate steps (with the non-MM synthesis term for an intermediate product such like protein) tends to make the oscillation be achieved more easily. This finding makes us realize that the transcription equation which contains the MM mRNA synthesis term is the fundamental part of a basic autoregulation model, while other intermediate steps (with non-MM synthesis) play the role of introducing slow dynamics for certain intermediate variables, as described in [8]. The above suggests that one may expect to compact any Goodwin model to a single equation for gene transcription like Eq. (9).

Eq. (9) is a single ODE which reflects a continuous dynamical system. The increment of continuous state variable at the time point 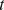 can be expressed in difference form:
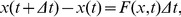
(10a)or in differential form:
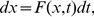
(10b)where 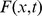 is the sum rate of synthesis rate minus degradation rate, and the time increment 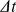 or 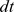 is an extremely tiny value. However, a recent molecular experiment shows that both continuous transcription and discontinuous transcription exist in yeast gene expression [12]. Moreover, more and more direct evidence show that the process of transcription for most genes is interspersed by “gene-on” and “gene-off” states by turns, in both prokaryotes and eukaryotes [13]–[15]. A silent interval (in which genes switch to “off” state and enter a refractory period) lying between two neighboring transcriptional events makes gene transcription not so continuous, and therefore, dynamics in such situation is described to be temporally discontinuous [15]. In this sense, if we remove those silent intervals, the presupposed continuous dynamical system of gene transcription is recovered.

**Figure 2:**
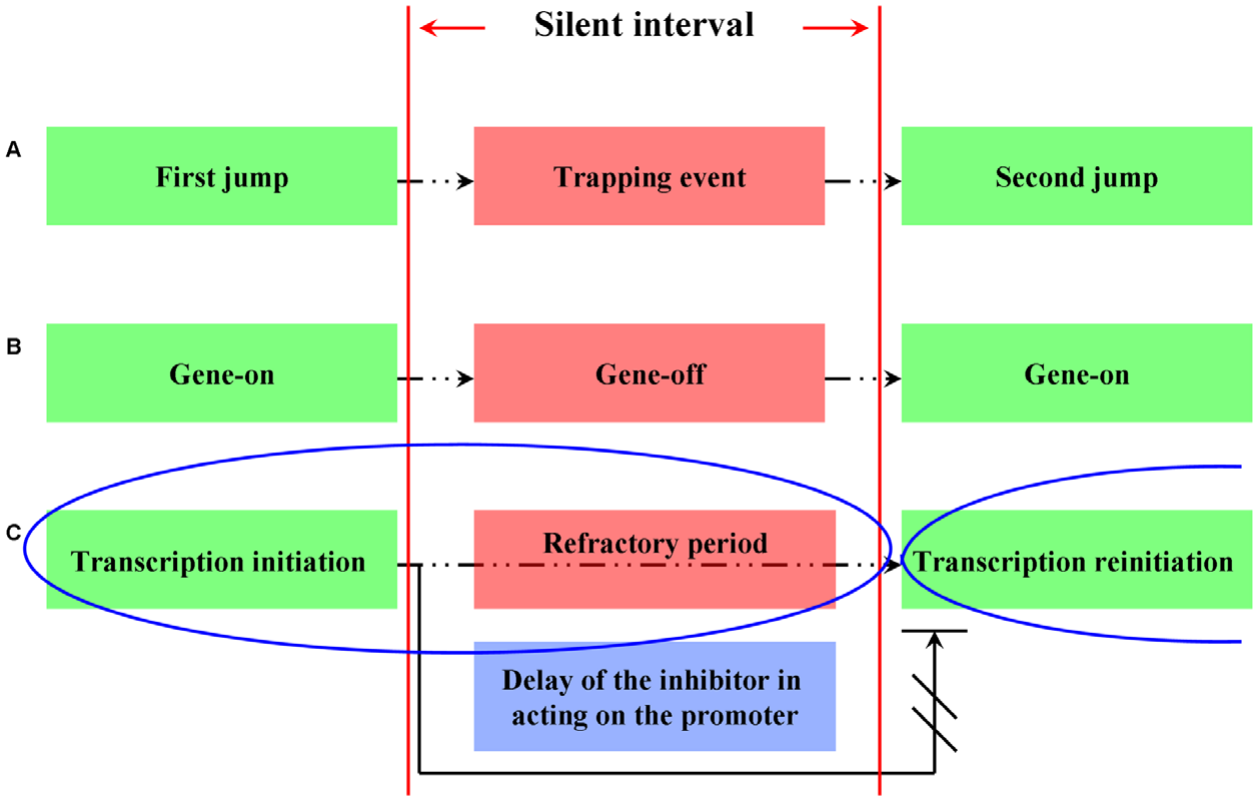
Consecutive events are separated by a silent interval. A. Inserting a trapping event into two neighboring jumps of the Brown particle in the continuous time random walk. B. The silent interval corresponds to the gene-off state. C. Temporally discontinuous transcription. The reinitiation will not launch until the refractory period is over, then the inhibitor starts to act on the promoter and triggers the reinitiation. Therefore, the time span of the silent interval can be treated to be equal to the time delay of the inhibitor generated in last transcriptional event. The blue ellipse indicates an intact transcriptional event.



In order to describe the temporally discontinuous gene transcription by using differential equations, we impose a silent interval upon its preceding transcriptional event. The schedule of an intact transcriptional event is then extended with a refractory period being appended as the end part, implying a relative slow transcriptional dynamics for that the total running time of an intact transcriptional event increases. With this treatment, two neighboring transcriptional events can be connected smoothly and continuously without an interval (Fig. 2). The idea of inserting silent intervals into continuous events can be traced back to the case where a trapping event (the Brown particle is temporarily immobile) is inserted into two neighboring jumps of continuous time random walk (Fig. 2) and eventually leads to fractional dynamics [16]–[19]. Different from this stochastic dynamics whose solution is usually described by transition probability, in our deterministic model, imposing a time interval upon a transcriptional event and increasing the running time of an intact transcriptional event will make each variable increment 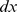 occur within a larger time increment rather than within the original 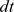. Since the original 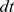 is an extremely tiny value (e.g. 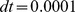 in dimensionless form), we take Jumarie’s notion of time increment 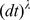, where 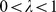 leads to 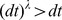 while 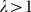 leads to 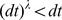 [20]. By setting 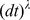 with 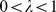 to be the larger time increment, we obtain the analog of Eq. (10b) which reads.

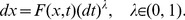
(11)The relation between the fractional difference and the finite difference has been given as
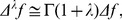
(12a)or in differential form:
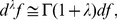
(12b)where 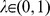 [20]–[22]. Multiplying both sides of Eq. (11) by 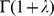 and taking account of Eq. (12b), we obtain



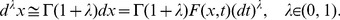
(13)Hence, with time evolving, we have a 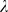-order differential equation:
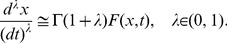
(14)The symbol of 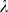-order derivative 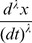 makes one recall the age-old issue presented in the communications between L’Hôspital and Leibniz: what if the order is 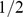 (see [23] and the Preface of [10]). Mathematicians have been inspirited by this story for over 300 years and their endeavors have led to a variety of nonequivalent definitions for the fractional order derivatives. By taking Caputo’s definition of the left-side fractional derivative and normalizing the constant coefficient 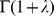 in Eq. (14) to unity, we obtain the generalized form:
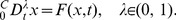
(15a)From Eq. (5) we know that 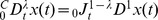. If 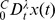 is differentiable, by calculating 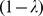-order derivatives of the both sides of Eq. (15a), we can obtain an equivalent equation:
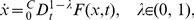
(15b)where 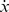 represents the first order derivative of 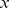. Different from the traditional ordinary differential equation 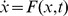 in which the product rate 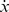 is locally determined by 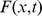, Eq. (15) shows long-term memory because the fractional derivative operator is non-local. Take 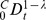 in Eq. (15b) for example, according to Eq. (5) and the commutative property of convolution, we know that



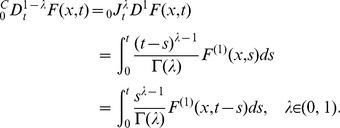
(16)Since 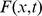 is the sum of synthesis rate minus degradation rate, we define the first order derivative 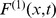 as the “acceleration of product gain”. The convolution kernel 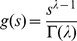 is used to weight 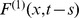 from nonce to remote history with 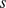 increasing from zero to current time point 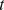. As depicted in Fig. 1, with normal timescale (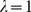), weights are always equal, implying that each transcriptional event launches *de novo*. Under this condition, the traditional ordinary differential equation 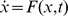 is recovered. In the case where 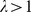, the farthest transcriptional event gives the highest impact while the weight given by the nearest transcriptional event is nearly close to zero. This case is in contradiction with regular physiological phenomena. Besides, 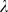 can be infinitely large under the condition of 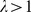. Therefore, we exclude 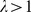 in our study. In contrast, the case in which 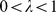 shows the property of “fading memory”. With such memory, a current event carries information form the preceding events, especially the nearest one. This property seems to be consistent with the observed phenomenon that transcriptional reinitiation is more common than *de novo* initiation in a discontinuously-transcribed gene [13]. However, the mechanisms of such transcriptional memory are not very clear by now. A feasible explanation may be that the loop scaffold forming in gene transcription would retain certain enzymes (or regulatory factors) of the preceding transcriptional events [24], [25] and sterically hinder the new recruited enzymes to take their place. Therefore, a subsequent transcriptional event remembers the preceding transcriptional events and takes a reinitiation rather than initiating *de novo*. The likelihood of reinitiation diminishes as the interval time elapses, and such relation can fit a simple exponential decay function [13]. Since 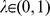, we simply use a regular decay expression 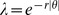 to represent the probability for a gene still “surviving” with the capability of transcriptional reinitiation after a time length 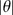 (time span of the silent interval), while 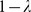 is the probability for the gene decaying to *de novo* initiation. The unknown system constant 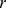 represents the probability per unit time for a still “surviving” gene to decay [26]. In this way, a relative small 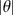 indicates a relative large 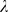, resulting in a relative small 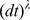. If the silent interval does not exist, namely, in the limit as 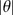 approaches zero, 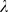 will approaches one and 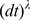 will approaches 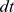, leading to the recovery of the early stage models for the continuous transcription.

### Numerical Studies of Fractional Dynamics in two Illustrative Examples – a minimal goodwin oscillator and a Rössler Oscillator

The first example is the Goodwin oscillator which was introduced originally in 1950s to simulate physiological oscillations in a closed loop with negative feedback.

**Figure 3:**
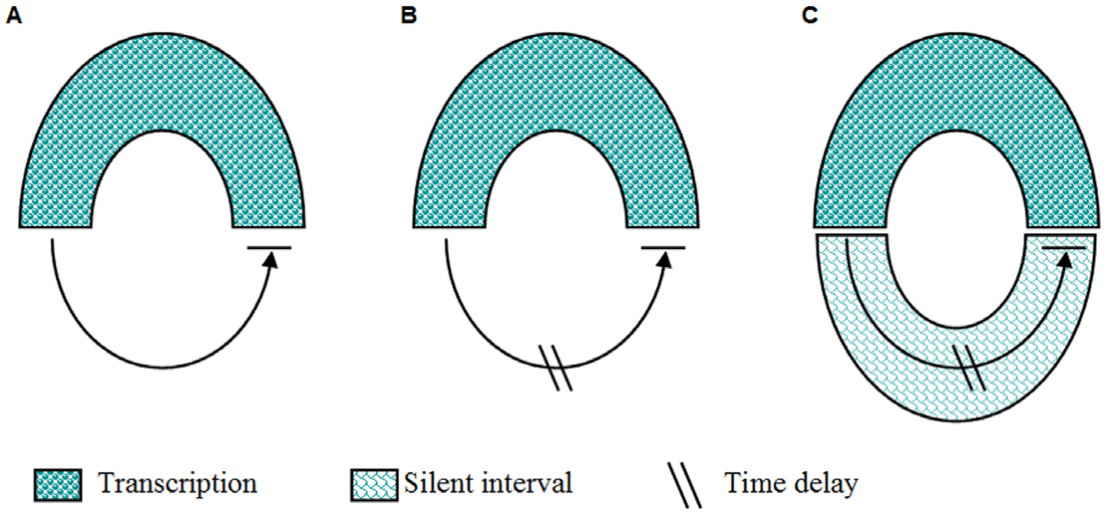
Different types of one-variable Goodwin model. A. Goodwin model with regular timescale. B. Goodwin oscillator with explicit time delay. C. The fractional delay Goodwin oscillator described by Eq. (17). The silent interval hinders the product to trigger the reinitiation of gene transcription and hence the time span of the silent interval is equal to the delay time.



The one-variable Goodwin model is expressed as Eq. (9). This model reflects by default a fast dynamics in which the products will be put into the feedback loop very quickly and then participate in the reactions immediately (Fig. 3A). However, this model does not generate sustained oscillation [6]. When the explicit time delay is introduced for generating sustained oscillation, a slow dynamics is achieved and the model becomes a delay Goodwin oscillator (Fig. 3B). If transcriptional discontinuity is considered, by involving explicit time delay and the deduced Eq. (15a), we obtain a “fractional delay Goodwin oscillator” which reads

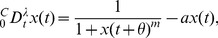
(17)where 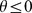 is the time delay and the symbol 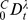 with 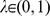 denotes the Caputo fractional derivative operator. Since both the time delay and the fractional operator are non-local, Eq. (17) describes a “dual memory” system.

In this model, we assume that the silent interval retards the inhibitor produced in a transcriptional event to act on the gene promoter until the transcriptional reinitiation starts. Under this assumption, the time delay can be set to be equal to 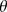 (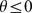) when 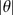 denotes the time span of the silent interval (Fig. 2C and Fig. 3C).

In order to avoid negative solution when time delay is involved in the Goodwin oscillator [27], the conditions required for non-negative solution should be established. Since the Goodwin oscillator can often be developed into several variants (e.g. the degradation term 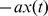 can be replaced by an MM expression [7], [28]), we investigate the existence and the uniqueness of the non-negative solution for the generalized form of the fractional delay Goodwin oscillator (see Appendix S1).

**Figure 4:**
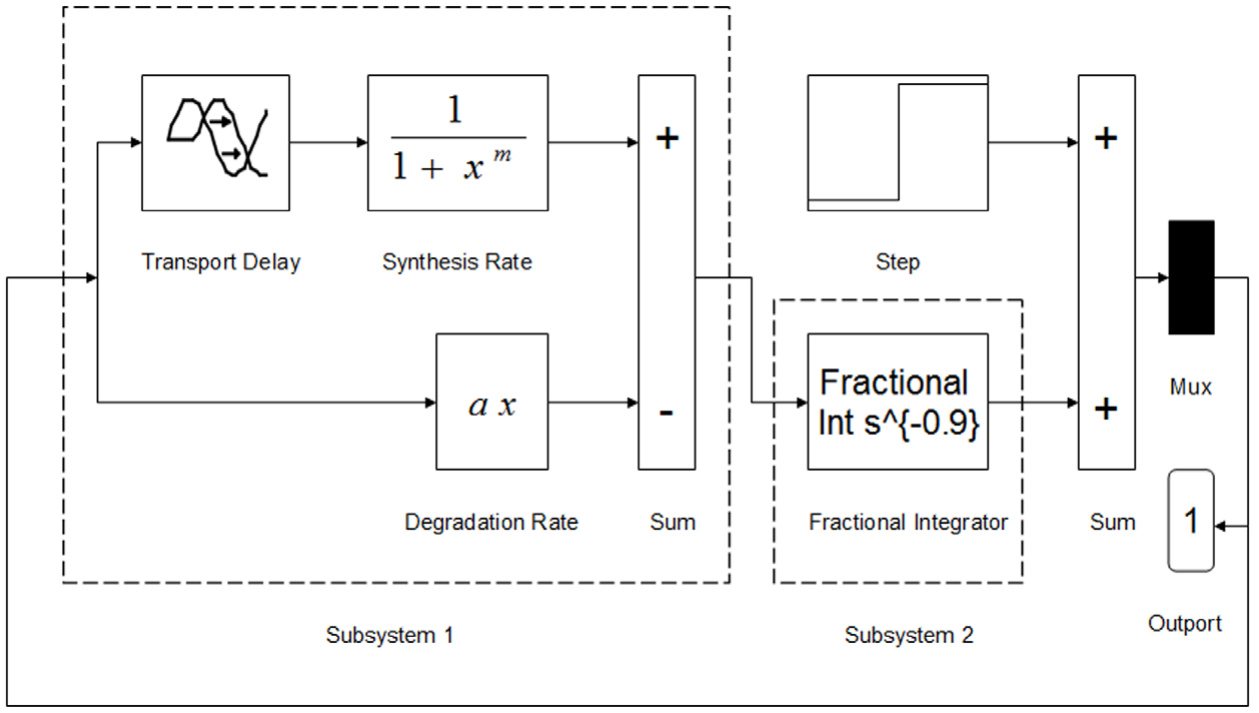
Simulink block diagram of the fractional delay Goodwin oscillator. Subsystem 1 containing a block of Transport Delay possesses the time lag effect, while subsystem 2 is a fractional integrator (with order of 0.9) which reflects the fading memory effect. The Step block is used for assigning the initial value in the beginning step, and the Outport block is used for collecting the simulation results.

The Simulink block diagram of Eq. (17) is depicted in Fig. 4. The fractional integrator used in this section is the well-established Oustaloup recursive filter which has been proved to fit well to the fractional operators and has been widely used in control systems [29]–[31]. It is suggested that because the same orders of the numerator and the denominator in the ordinary Oustaloup filter may cause algebraic loops in simulation, a low-pass filter must be appended to the Oustaloup filter to avoid such disadvantage; the stiff equation solver *ode23tb* is selected to ensure high efficiency and accuracy [32]. In simulation, we fix the parameter 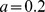 in the degradation term.

The second example is the Rössler oscillator which is originally used to study chaotic kinetics in biochemical system [33]. Novak and Tyson [7] used this model to describe the activator amplification with two negative feedback loops in parallel. From the viewpoint of mathematical modeling, the common phenomenon of coupling different genetic oscillator motifs in gene regulation would tend to cause complex non-linear oscillations like chaos. However, the deterministic chaos has not been detected more often in real data from experiments, and Novak and Tyson speculated that such chaos might be swamped by white noise and averaged out in large populations of cells [7]. This speculation is passable if stochastic factors are introduced. However, since all we have discussed so far are deterministic models, can we give an alternative explanation about the lack of chaos in gene regulation merely from the angle of determinism?

For convenience, we use the classic Rössler system (the “model of a model”; [34])
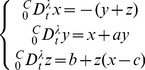
(18)to illustrate how the globally slow transcription affects the system’s dynamical behavior. The parameters are set by 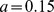, 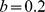 and 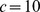 with sampling period 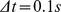 for time series, according to the reference [35]. Because of the global property of 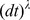, all three individual equations of Eq. (18) share a commensurate order just like the way given by the traditional commensurate first order Rössler system (but with 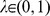). The numerical method for fractional calculus is the Oustaloup recursive filter mentioned above. The largest Lyapunov exponent (LLE) is calculated by using the method of Rosenstein et al. [35], and the embedded delay and dimension are evaluated by using the methods of Kim et al. [36] and Kugiumtzis [37].

### Results and Discussion

By inserting silent intervals into consecutive transcriptional events and globally replacing 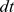 by a larger increment 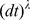, we make the early stage models change from ODE to FDE. The impact of the order fractionization on genetic oscillation can be shown by the Goodwin oscillator and the Rössler oscillator.

**Figure 5:**
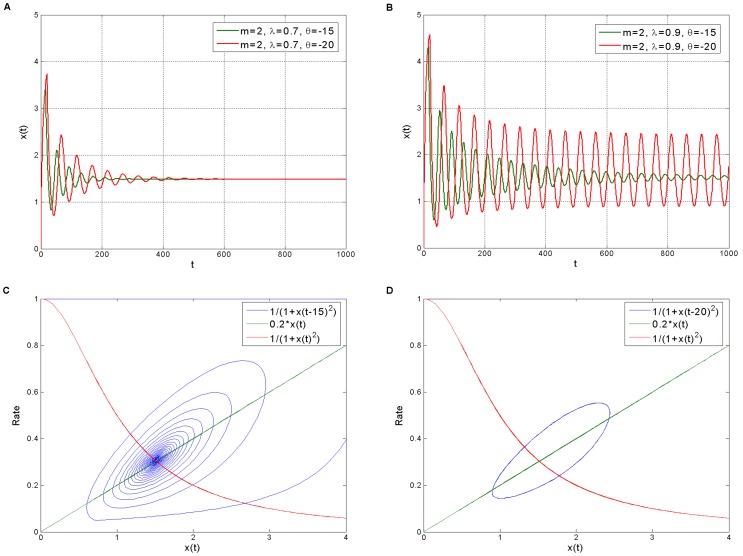
Numerical results of the fractional delay Goodwin oscillator. A. A comparison of the solutions of Eq. (17) with different time delay (–15 and –20, respectively) under the same condition of 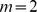, 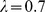 and 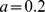. No sustained oscillations occur in both cases. B. Under the same condition of 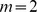, 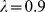 and 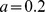, the solution of Eq. (17) with 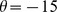 indicates a damped oscillation on the way to steady state, while the case with 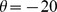 corresponds to sustained oscillation. C. Plots of the synthesis rate (without delay), the degradation rate and the rate of synthesis containing delay, against the present value 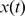. The blue curve indicates the damped oscillation. D. Plots of the synthesis rate (without delay), the degradation rate and the rate of synthesis containing delay, against the present value 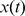. The blue circle indicates sustained oscillation.

For the fractional delay Goodwin oscillator, the numerical results show that no sustained oscillations occur when 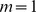; however, with fixed 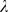 and 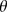, the increase of 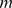 leads to the destabilization of the steady state (data not shown). In the case of 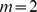 (Fig. 5A and Fig. 5B), when 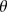 is specified, the increase of 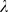 leads to the stability loss. The impact of time delay is clearly shown in Fig. 5B–5D. When 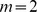 and 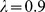, setting the delay time 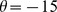 generates damped oscillation which is on the way to steady state, while 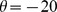 produces periodical sustained oscillation. In this case, by plotting the rate of degradation or synthesis versus the present values 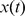, the trajectory of the time-delayed synthesis rate with 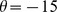 is attracted to the intersection point (the steady state of 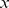) of the degradation rate and the non-delayed synthesis rate (Fig. 5C). With an extending delay of 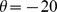, the time-delayed loop overshoots and undershoots the steady state, indicating the periodical sustained oscillation of 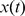 (Fig. 5D).

Novak and Tyson [7] proposed four general requirements for oscillation in gene regulation: (1) negative feedback loop; (2) sufficient nonlinearity; (3) sufficient time delay; (4) proper balancing of timescale (namely, 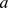 in the degradation term of Eq. (17) must not be too large). However, by introducing fractional dynamics, the decrease of the order 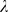 would tend to make the oscillation more stable, even though a sufficient time delay is reached. This can be clearly shown in Fig. 5A and Fig. 5B: when sufficient nonlinearity (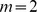), sufficient time delay (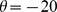) and proper balancing of timescale (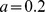) are satisfied, the decrease of 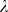 (from 0.9 to 0.7) makes the oscillatory limit cycle become a fixed point attractor. Since the order 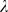 (as shown in Fig. 1) can be used to indicate the strength of memory in fractional dynamics, we suggest the use of the term “sufficient memory” for the 3rd requirement rather than using only “sufficient time delay” when fractional dynamics is involved.

**Figure 6:**
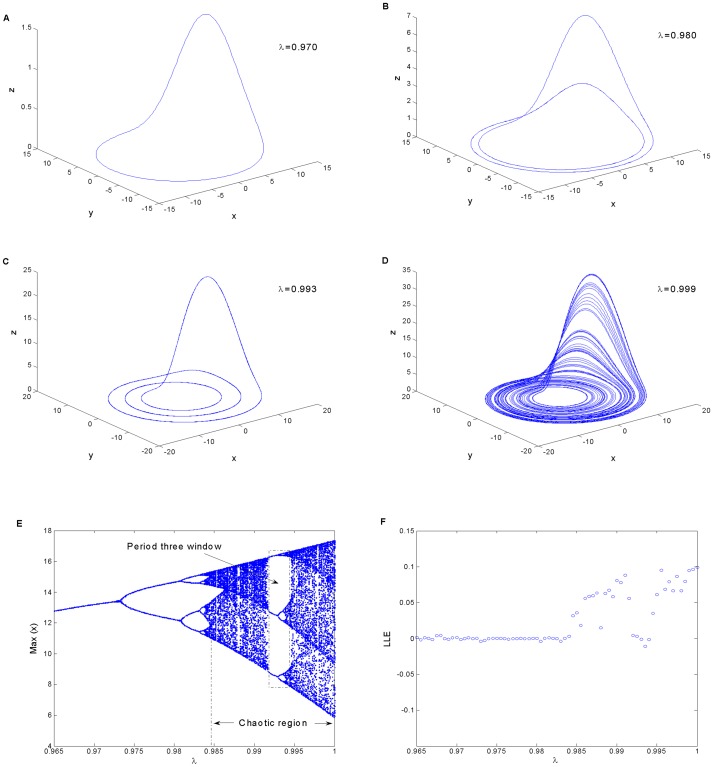
The Rössler oscillator running under globally slow transcriptional dynamics. A. Period one motion. B. Period two motion. C. Period three motion. D. Chaotic motion. E. Diagram of period doubling bifurcation. F. Plot of the largest Lyapunov exponent against the system orders. LLE indicates chaotic motion, limit cycle (or quasiperiodic motion) and fixed point attractor with positivity, zero and negativity, respectively.

For the Rössler oscillator, the traditional commensurate first order model shows the classic unimodal folded chaos with LLE of +0.0995 (Fig. 6F). When fractional dynamics is involved, it is clearly shown that the dynamical behavior changes from non-periodic (chaotic) motion (LLE>0) to periodic motion (LLE = 0) with 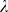 decreasing (Fig. 6F). Some points that seem to be outliers at around 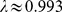 (Fig. 5F) are attributed to the meeting of the period-three window which is embedded in the chaotic region (Fig. 6C and Fig. 6E). The existence of the period three window in the diagram of period doubling bifurcation (Fig. 6E) implies that the chaotic dynamics of the fractional Rössler model can be interpreted through the Sharkovskii order [38] or Li-Yorke theorem [39]. The critical value for the route to chaos is 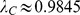, and the region corresponding to the order interval 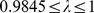 is defined as the chaotic region. Therefore, if we simply consider a uniform distribution of 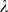 within the order interval 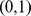, the fractional Rössler oscillator with originally specific parameters will show a probability of more than 0.98 to behave non-chaotically. This deterministic fractional dynamics may provide an alternative explanation for understanding why the deterministic chaos in gene regulation has not been detected more often in real data from experiments [7].

**In conclusion**, a transcription equation with a term representing MM mRNA synthesis is the core of a basic autoregulation model. Therefore, any basic autoregulation circuit can be minimized to such a single equation. Traditionally, slow dynamics can be achieved via setting explicit time delay to the state variable of a minimal model. In this study, we propose a globally slow transcription on the basis of the recent observation that discontinuous transcription may be more common than continuous transcription. By inserting silent intervals into neighboring transcriptional events which are presupposed to be continuous, and appending an inserted silent interval to the end of the preceding event, we establish that the running time for an intact transcriptional event increases. By globally replacing the original time increment for each state increment by a larger one, we obtain a fractional model for gene transcription. With the assumption that the silent interval hinders the product to trigger the reinitiation of gene transcription and hence causes time delay, we construct a fractional delay Goodwin oscillator. Since both time delay and fractional operator are non-local, such new type of Goodwin oscillator is a “dual memory” system. To avoid negative solution when time delay is involved, the existence and the uniqueness of the non-negative solution for the generalized form of the fractional delay Goodwin oscillator are also studied. The numerical studies show that the explicit time delay tends to destabilize the steady state, while the fractionization of the order tends to make the system stable. This result makes us realize that the requirement “sufficient time delay” for genetic oscillation is not sufficient and should be changed to “sufficient memory” when fractional dynamics is involved. When we examine another well-known genetic oscillator – the Rössler oscillator which describes the activator amplification coupled with two negative feedback loops in parallel, the diagram of period doubling bifurcation against the orders reveals that the globally slow dynamics induced via discontinuous transcription tends to lower the chance of a coupled or more complex nonlinear genetic oscillatory system behaving chaotically.

## Supporting Information

Appendix S1 **Existence and uniqueness of the non-negative solution for the generalized form of the fractional delay Goodwin oscillator.**
(DOC)
