# Supplementary material for: Fractional Dynamics of Globally Slow Transcription and Its Impact on Deterministic Genetic Oscillation
Source: PLoS One. 2012 Jun 5;7(6):e38383. doi: 10.1371/journal.pone.0038383 (PMC3367935; doi:10.1371/journal.pone.0038383)
Supplement: Appendix S1 — Existence and uniqueness of the non-negative solution for the generalized form of the fractional delay Goodwin oscillator. (DOC) [file pone.0038383.s001.doc]

**APPENDIX: Existence and uniqueness of the non-negative solution for the generalized form of the fractional delay Goodwin oscillator**

**1. Basic definitions and preliminaries for the functional analysis of the generalized fractional delay Goodwin oscillator**

To avoid the negative concentrations when time lag is involved in the Goodwin oscillator [1], it is important to establish that non-negative initial values give rise to non-negative solutions since most differential equations that arise in biology are intrinsically used for describing non-negative quantities.

We adopt the following notations for the retarded functional differential equations [2]: $R$ is an one-dimensional real Euclidean space with norm , and $C([a,b],R)$ is the Banach space of continuous functions mapping the interval $[a,b]$ into $R$ with the topology of uniform convergence. If $[a,b]=[-\tau,0]$, we denote a Banach space of continuous functions mapping the interval $[-\tau,0]$ into $R_{\geq0}$ by $C([-\tau,0],R_{\geq0})$, then for a given function $\varphi\in C([-\tau,0],R_{\geq0})$, the norm of $\varphi$ is defined as $\left\|\varphi\right\|=\sup_{-\tau\leq\theta\leq0} \left|\varphi(\theta)\right|$.

For the Eq. (17), we define $x_{t}(\theta):=x(t+\theta)$, then the present state $x(t)$ can be represented by $x_{t}(0)$, where $\theta=0$ denotes no delay. Then, Eq. (17) can be rewritten as

$${}_{0}^{C}D_{t}^{\lambda}x(t)=\frac{1}{1+x(t+\theta)^{m}}-ax(t)=\frac{1}{1+x_{t}(\theta)^{m}}-ax_{t}(0). (A1)$$

It is obvious that the right side of the equal sign in Eq. (A1) is about only one variable $x_{t}$. Therefore, following the way suggested by Smith [3], the initial value problem about Eq. (A1) can be generally expressed as

$$\left\{\begin{matrix} {}_{0}^{C}D_{t}^{\lambda}x(t)=f(t,x_{t}), t\in(0,T], \\ x(t)=\varphi(t)\geq0, t\in[-\tau,0], \end{matrix} (A2)\right.$$

where $\lambda\in(0, 1)$, $\varphi\in C([-\tau,0],R_{\geq0})$, the positive constant $\tau$ is the maximum time delay of the system, and $t$ in the given continuous nonlinear function $f(t, \cdot)$ is the evolving time variable of a dynamical system (whatever implicit or explicit). The function of time $x(t)$ is a solution of Eq. (A2) starting at $x(0)$.

From the viewpoint of signals and systems, we treat $x_{t}$ in $f(t, x_{t})$ as the input signal, and $x(t)$ in ${}_{0}^{C}D_{t}^{\lambda}x(t)$ as the output signal; then, according to the concepts of “interconnections of systems” and “systems with memory” [4], Eq. (A2) can be illustrated as a “dual memory” system with cascade interconnection of two subsystems. The subsystem 1 containing a transport delay block reflects the discrete delay memory, while the subsystem 2 which is a fractional integrator reflects the “long-term memory effect” (Fig. 4). In the iterative process of simulation, if the non-negative initial value of the input signal at $t=0$ generates a non-negative output $x(t=0)$, then the value of $x(t=0)$ will serve as the next input signal at $t=0+h$ ($h$ is the time step size). In this sense, the requirement of non-negative initial values giving rise to non-negative solutions means that all non-negative input signals $x_{t}$ must generate non-negative outputs. Therefore, in Eq. (A2), $x_{t}\in C([-\tau,0],R_{\geq0})$. Since $\lim_{\lambda\to0} {}_{0}J_{t}^{\lambda}f(t)=f(t)$ [5], in the limit as $\lambda$ approaches zero, we have $x(t)$ approaches $f(t,x_{t})$. Therefore, $f(t,x_{t})\geq0$ is necessary for $x(t)\geq0$. By setting a time interval $I=[0,T]$ which includes the initial time point $t=0$, we have the continuous function $f:I\times C\to R_{\geq0}$ with $\lim_{t\to0^{+}} f(t,x_{t})=+\infty$ (i.e., $f$ is singular at $t=0$). In next subsection, we will investigate the existence and the uniqueness of the non-negative solution to Eq. (A2), relying on the Leray-Schauder alternative theorem in a cone, the Banach fixed point theorem and the Arzela-Ascoli theorem.

**Definition A** (Cone and partial ordering; [6]) Let $X$ be a Banach space. A cone $K\subset X$ is a closed convex set with $\alpha K\subset K$ for all $\alpha\geq0$ and $K\cap(-K)=\left\{0\right\}$. A partial ordering $\leq$ with respect to $K$ is defined by $x\leq y$ iff $y-x\in K$.

**Definition B** (Completely continuous operator; [7]) Consider two Banach spaces $X$ and $Y$, a subset $\Omega$ of $X$ and a map $F:\Omega\to Y$. $F$ is said to be a completely continuous operator if it is continuous and maps bounded subsets of $\Omega$ into relatively compact sets.

**Theorem** **A** (Leray-Schauder alternative theorem; [8]) Let $E$ be a Banach space, $C$ a convex subset of $E$, and assume $0\in C$. Let $F:C\to C$ be a completely continuous operator, and let

$$\varepsilon(F)=\left\{x\in C|x=\sigma F(x)\right\}$$

for some $0<\sigma<1$. Then either $\varepsilon(F)$ is unbounded, or $F$ has a fixed point.

**Theorem** **B** (Banach fixed point theorem; [9]) Let $X$ be a Banach space, $D\subset X$ closed and $F:D\to D$ a strict contraction, i.e. $\left|F(x)-F(y)\right|\leq L\left|x-y\right|$ for certain Lipschitz constant $L\in(0, 1)$ and all $x,y\in D$. Then $F$ has a unique fixed point $z_{*}$.

**Theorem** **C** (Arzela-Ascoli theorem; [10]) Let $\Omega$ be an open bounded subset of $R^{n}$, and the space of continuous real-valued functions on $\bar{\Omega}$ denoted by $C(\bar{\Omega})$ be a Banach space under the norm $\left\|u\right\|=\sup_{x\in\bar{\Omega}} \left|u(x)\right|$. A subset $K\subset C(\bar{\Omega})$ is equicontinuous provided for every $\varepsilon>0$ there is a $\delta>0$ such that $\left\|x_{1}-x_{2}\right\|<\delta$ implies $\left|u(x_{1})-u(x_{2})\right|<\varepsilon$ for every $x_{1},x_{2}\in\Omega$ and every $u\in K$. A subset of $C(\bar{\Omega})$ is relatively compact iff it is bounded and equicontinuous.

**Theorem** **D** (Cantor-Heine theorem; [11]) A function that is continuous on a closed interval is uniformly continuous on that interval.

**2. Existence of non-negative solution**

The Caputo fractional derivative operator shows advantage in dealing with initial condition. If the fractional derivative ${}_{0}^{C}D_{t}^{\lambda}f(t)$ is integrable, according to the equality (2.4.44) in the reference [12], we have

$${}_{0}J_{t}^{\lambda}({}_{0}^{C}D_{t}^{\lambda}f(t))=f(t)-f(0), (A3)$$

where $\lambda\in(0, 1)$. For the first equation in (A2), we have

$${}_{0}J_{t}^{\lambda}({}_{0}^{C}D_{t}^{\lambda}x(t))={}_{0}J_{t}^{\lambda}f(t,x_{t}), t\in(0,T]. (A4)$$

According to Eq. (A3), we get

$$x(t)=x(0)+{}_{0}J_{t}^{\lambda}f(t,x_{t}), t\in(0,T]. (A5)$$

Therefore, Eq. (A2) is equivalent to

$$x(t)=\left\{\begin{matrix} x(0)+{}_{0}J_{t}^{\lambda}f(t,x_{t}), t\in(0,T], \\ \varphi(t)\geq0, t\in[-\tau,0]. \end{matrix} (A6)\right.$$

Let $y(\cdot):[-\tau,T]\to R_{\geq0}$ be a function defined by

$$y(t)=\left\{\begin{matrix} \varphi(0), t\in I, \\ \varphi(t)\geq0, t\in[-\tau,0], \end{matrix} (A7)\right.$$

where $I\in[0, T]$. For each $z\in C(I, R)$ with $z(0)=0$, we denote by $\bar{z}$ the function defined by

$$\bar{z}(t)=\left\{\begin{matrix} z(t), t\in I, \\ 0, t\in[-\tau,0]. \end{matrix} (A8)\right.$$

Now $x(\cdot)$ can be decomposed as $x(t)=\bar{z}(t)+y(t)$, $t\in[-\tau, T]$, while $x_{t}=\bar{z}_{t}+y_{t}$ for $t\in I$. Hence, by (A6), the system (A2) is equivalent to

$$z(t)={}_{0}J_{t}^{\lambda}f(t,\bar{z}_{t}+y_{t}), t\in I. (A9)$$

From the viewpoint of signals and systems, $\bar{z}_{t}+y_{t}$ in (A9) is the input signal which contains the information of initial value, while $z(t)$ is the output of the system.

Let $\Omega=\left\{z\in C(I, R)\left|z(0)=0\right.\right\}$ be the Banach space endowed with the norm as $\left\|z\right\|=\sup_{0\leq t\leq T} \left|z(t)\right|$, $z\in\Omega$, and $K=\left\{z\in\Omega\left|z(t)\geq0, t\in I\right.\right\}$ be a cone of $\Omega$. Define an operator $F:K\to K$ by

$$Fz(t):={}_{0}J_{t}^{\lambda}f(t,\bar{z}_{t}+y_{t}), t\in I. (A10)$$

**Theorem** **E** Let $f(t,x_{t})$ be a non-negative continuous function with $t\in(0, T]$, $x_{t}\in C([-\tau, 0], R_{\geq0})$ and $\lim_{t\to0^{+}} f(t,x_{t})=+\infty$. If $f(t,x_{t})$ is continuous on $[0, T]\times[0, +\infty)$, then the Eq. (A2) has a non-negative solution $x_{*}$.

**Proof**. We will follow the “4-step procedure” [13,14] to finish this proof.

*Step 1*. In this step, we will show that $F$ is continuous.

Let $G\subseteq K$ be bounded, then we set $\left\|z\right\|=c$ for all $z\in G$. Since $x_{t}=\bar{z}_{t}+y_{t}$ and $\bar{z}=z$ with $t\in I$, by the assumptions of the continuity of $f(t,x_{t})$ on $I\times[0, +\infty)$ in **Theorem** **E**, we know that $f(t,\bar{z}_{t}+y_{t})$ is continuous on $I\times[0, c+\left\|\varphi\right\|]$ when $c+\left\|\varphi\right\|<+\infty$. Further, $f(t,\bar{z}_{t}+y_{t})$ is uniformly continuous, according to the Cantor-Heine theorem. Since a uniformly continuous mapping is bounded [15], we set $M=\max f(t,\bar{z}_{t}+y_{t})$, $\forall z\in G$. For $\varepsilon>0$ and any $t,t+\varepsilon\in[0, T)$, applying Newton-Leibniz formula, we have

It is obvious that

;

a similar result is obtained for when $t=T$. Then, by the arbitrariness of $t\in I$, ${}_{0}J_{t}^{\lambda}f(t,\bar{z}_{t}+y_{t})$ is continuous on time domain, indicating $Fz(t)$ is continuous on $I$.

Since $f(t,\bar{z}_{t}+y_{t})$ is uniformly continuous on $I\times[0, c+\left\|\varphi\right\|]$, for two arbitrary elements in $G$ at any same time point, e.g. $z_{1}(t),z_{2}(t)\in[0, c]$, $\forall\varepsilon>0$, $\exists\delta>0$ such that

,

whenever $\left|z_{1}(t)-z_{2}(t)\right|<\delta$ for $t\in I$. As a result,

proving the continuity of $F$ on $G$.

*Step 2*. $F$ maps bounded sets of $K$ into bounded sets in $K$.

For each $z\in G$, we have

Therefore, $F(G)$ is bounded.

*Step 3*. We will show that $F$ is equicontinuous in this step.

For a single element $F(z_{1})\in F(G)$ and $\Delta t>0$, let $0\leq t<t+\Delta t<T$ and $z_{1}(t)$, $z_{1}(t+\Delta t)$ represent two values in the orbit of $z_{1}$ at different time point. Since $z_{1}\in C(I, R_{\geq0})$, where $C(I, R_{\geq0})$ is the Banach space of continuous functions mapping the interval $[0, T]$ into $R_{\geq0}$, we known $z_{1}$ is uniformly continuous on time domain. Therefore, $\forall\delta>0$, $\exists\hat{\delta}>0$ such that $\left|z_{1}(t)-z_{1}(t+\Delta t)\right|<\delta$ whenever $\left|\Delta t\right|<\hat{\delta}$. Thus, we have

Let $\varepsilon=\frac{2T^{\lambda}M}{\Gamma(\lambda+1)}$, we choose $\hat{\delta}=T=\left(\frac{\varepsilon\Gamma(\lambda+1)}{2M}\right)^{\frac{1}{\lambda}}$. Then whenever $\left|\Delta t\right|<T$, there exists $\left|z_{1}(t)-z_{1}(t+\Delta t)\right|<\delta$ such that $\left|Fz_{1}(t)-Fz_{1}(t+\Delta t)\right|<\varepsilon$. Therefore, $F(z_{1})$ is uniformly continuous. By the arbitrariness of $F(z_{1})\in F(G)$, $F(G)$ is equicontinuous. According to the Arzela-Ascoli theorem, $F(G)$ is relatively compact. Since the continuous operator $F$ maps the bounded set $G$ into the relatively compact set $F(G)$, we known $F$ is a completely continuous operator.

*Step 4*. Let $\varepsilon(F)=\left\{z\in K\left|z=\sigma F(z)\right.\right\}$ for some $0<\delta<1$. Similar to (A13), we obtain

Since $\sigma\in(0, 1)$ and $\left|Fz(t)\right|=0$ is allowed, for $\varepsilon(F)$ we have

$$\begin{matrix} \left|z(t)\right|=\sigma\left|Fz(t)\right| \\ \leq\left|Fz(t)\right| \\ \leq\frac{MT^{\lambda}}{\Gamma(\lambda+1)}. (A15) \end{matrix}$$

If we consider

$$h=\max\frac{MT^{\lambda}}{\Gamma(\lambda+1)}+o, (A16)$$

where $o$ represent an arbitrary small positive value, then any solution of $z=\sigma F(z)$in $\varepsilon(F)$ satisfies $\left\|z\right\|<h$. Therefore, $\varepsilon(F)$ is bounded. According to **Theorem** **A**, $F$ has a fixed point $z_{*}\in G$, satisfying

$$z_{*}(t)=Fz_{*}(t)={}_{0}J_{t}^{\lambda}f(t,\bar{z}_{*,t}+y_{t}), t\in I, z\geq0, (A17)$$

from which we know Eq. (A9) has a non-negative solution $z_{*}$. Therefore, there exists a non-negative solution $x_{*}$ of Eq. (A2), satisfying $x_{*}(t)=z_{*}(t)+\varphi(0)$ for $t\in I$. Moreover, if $\varphi(0)>0$, $x_{*}$ is a strictly positive solution. The proof is completed.

**3. Unique existence of solution**

In this section we give conditions which render unique non-negative solution to (A2).

**Theorem** **F** Let $f(t,x_{t})$ be a non-negative continuous function with $t\in(0,T]$, $x_{t}\in C([-\tau,0], R_{\geq0})$ and $\lim_{t\to0^{+}} f(t,x_{t})=+\infty$. If $f(t,x_{t})$ is continuous on $[0, T]\times[0, +\infty)$, let $f(t,x_{t})$ be Lipschitz with respect to the second variable with Lipschitz constant $l>0$, say, $\left|f(t,x_{1,t})-f(t,x_{2,t})\right|\leq l\left|x_{1}-x_{2}\right|$. If $\exists L=\frac{T^{\lambda}l}{\Gamma(\lambda+1)}<1$, then Eq. (A2) has unique non-negative solution $x_{*}$.

**Proof**. From above section we have known that the solution of (A9) is equivalent to the fixed point of the operator $F$ defined on $K$. Hence, for $z_{1},z_{2}\in K$ and $t\in I$, we have

According to Banach fixed point theorem, $F$ has unique fixed point in $K$, indicating the uniqueness of non-negative solution $x_{*}$ of Eq. (A2). The proof is completed.

**References**

1. MacDonald N (1977) Time lag in a model of a biochemical reaction sequence with end product inhibition. J Theor Biol 67: 549-556.

2. Kuang Y (1993) Delay Differential Equations with Applications in Population Dynamics. New York: Academic Press.

3. Smith H (2011) An Introduction to Delay Differential Equations with Applications to the Life Sciences. Springer Science & Business Media, LLC. pp. 31.

4. Oppenheim AV, Willsky AS, Nawab SH (1997) Signals and Systems, 2nd Edition. Prentice Hall.

5. Podlubny I (1999) Fractional Differential Equations. San Diego: Academic Press. pp. 65.

6. Deimling K (1985) Nonlinear functional analysis. New York: Springer-Verlag. pp. 218.

7. Deimling K (1985) Nonlinear functional analysis. New York: Springer-Verlag. pp. 55.

8. Granas A, Dugundji J (2003) Fixed Point Theory. New York: Springer-Verlag. pp. 124.

9. Deimling K (1985) Nonlinear functional analysis. New York: Springer-Verlag. pp. 39.

10. Granas A, Dugundji J (2003) Fixed Point Theory. New York: Springer-Verlag. pp. 607.

11. Zorich VA (2004) Mathematical Analysis, I. Springer-Verlag. pp. 164.

12. Kilbas AA, Srivastava HM, Trujillo JJ (2006) Theory and Applications of Fractional Differential Equations. Amsterdam: Elsevier. pp. 96-90.

13. Babakhani A, Enteghami E (2009) Existence of positive solutions for multiterm fractional differential equations of finite delay with polynomial coefficients. Abstr Appl Anal 2009: 768920.

14. Liao C, Ye H (2009) Existence of positive solutions of nonlinear fractional delay differential equations. Positivity 13: 601-609.

15. Berger MS (1977) Nonlinearity and functional analysis. New York: Academic Press. pp. 65.
